# Wnt signaling modulator DKK4 inhibits colorectal cancer metastasis through an AKT/Wnt/β-catenin negative feedback pathway

**DOI:** 10.1016/j.jbc.2022.102545

**Published:** 2022-09-29

**Authors:** Junrong Liang, Lina Sun, Yujun Li, Wanning Liu, Danxiu Li, Ping Chen, Xin Wang, Juan Hui, Jinchi Zhou, Hao Liu, Tianyu Cao, Maogui Pang, Meng Guo, Xin Wang, Xiaodi Zhao, Yuanyuan Lu

**Affiliations:** 1 Department of Gastroenterology, Tangdu Hospital, Fourth Military Medical University, Xi’an, China; 2 State Key Laboratory of Cancer Biology, National Clinical Research Center for Digestive Diseases and Xijing Hospital of Digestive Diseases, Fourth Military Medical University, Xi’an, China; 3 Department of Gastroenterology, The Affiliated Children’s Hospital of Xi’an Jiaotong University, Xi’an, China; 4 Department of Endocrinology, The Second Affiliated Hospital of Xi’an Jiaotong University, Xi’an, China; 5 College of Life Sciences, Northwest University, Xi’an, China; 6 Department of Gastroenterology, Xingping People's Hospital, Xianyang, China; 7 Department of Gastroenterology, General Hospital of Northern Theater Command, Shenyang, China

**Keywords:** DKK4, Wnt pathway, AKT, colorectal cancer, metastasis, CRC, colorectal cancer, FZD6, Frizzled 6, HGF, hepatocyte growth factor, IHC, Immunohistochemistry, LV-DKK4, Lentivirus carrying the human DKK4 gene, qRT‒PCR, quantitative real-time PCR, siDKK4, small interfering RNAs against DKK4

## Abstract

Aberrant activation of the Wnt/β-catenin signaling pathway is implicated in most malignant cancers, especially in the initiation and progression of colorectal cancer (CRC). DKK4 is a classical inhibitory molecule of the Wnt/β-catenin pathway, but its role in CRC is ambiguous, and the molecular mechanism remains unclear. Here, we determined DKK4 expression was significantly upregulated in 23 CRC cell lines and 229 CRC tissues when analyzed by quantitative PCR and immunohistochemistry, respectively. Our analysis of tissue samples indicated the survival time of CRC patients with high DKK4 expression was longer than that of patients with medium-low DKK4 expression. We examined the effects of DKK4 on cell proliferation and metastasis by cell counting kit-8 assays, transwell assays, and subcutaneous and metastatic mouse tumor models, and we discovered that DKK4 silencing promoted the metastasis of CRC cells both *in vitro* and *in vivo*. Our RNA-seq analysis revealed that AKT2, FZD6, and JUN, which play important roles in AKT and Wnt signaling, were significantly increased after DKK4 knockdown. DKK4 represses Wnt/β-catenin signaling by repressing FZD6 and AKT2/s552 β-catenin in CRC. Further experiments revealed recombinant Wnt3a and LiCl could induce DKK4 expression. Moreover, our bioinformatics analysis and luciferase reporter assays identified posttranscriptional regulators of DKK4 in CRC cells. In summary, DKK4 is elevated in CRC and inhibits cell metastasis by a novel negative feedback mechanism of the Wnt3a/DKK4/AKT/s552 β-catenin regulatory axis to restrict overactivation of Wnt activity in CRC. Therefore, DKK4 restoration may be applied as a potential CRC therapeutic strategy.

Colorectal cancer (CRC), with more than 1.8 million new cases each year, is the third most frequent cancer globally. More than 861,000 patients die from CRC each year, which is the second leading cause of cancer death (1, 2). At the same time, the incidence and mortality of colorectal cancer are also increasing each year in China (3, 4). It is well known that early diagnosis and early surgical resection are generally considered to be the most effective ways to reduce CRC mortality. However, as the early symptoms of colorectal cancer are not obvious, an estimated 50% to 60% of CRC patients present with metastases at the time of diagnosis (5). Treatments of advanced CRC include palliative chemotherapy, targeted therapy, and immunotherapy, as well as local treatment. Nevertheless, the 5-years survival rate is only 14% (6). Therefore, the study of molecular mechanisms and new therapeutic targets for CRC is essential.

The Wnt signaling pathway plays an important role in the occurrence and development of colorectal cancer, and the Dickkopf (DKK)family is a classical family of inhibitory molecules in this pathway. Increasing evidence suggests that DKK family members play an important role in the occurrence and development of tumors. The gene family of DKKs encodes the secreted glycoprotein as inhibitory molecules of the typical Wnt signaling pathway, which consists of five members: DKK1-4 and DKKL1 (soggy) (7). DKK glycoproteins, except soggy, contain an N-terminal signaling peptide and two conserved cysteine-rich domains separated by linkers. The cysteine-rich amino-terminal domain (Cys-1) is unique to each DKK, while the carboxy-terminal cysteine-rich domain (Cys-2) is highly conserved among all DKKs. We previously found that miR-100 and miR-125b promoted Wnt signaling activity by targeting DKK1 and DKK3, leading to cetuximab resistance in CRC (8). DKK2 is a functional tumor suppressor that regulates the tumorigenesis of CRC by antagonizing Wnt/β-catenin signaling (9). DKK4 presents inconsistent expression and plays different roles in different cancers. In hepatocellular carcinoma, DKK4 expression is reduced, and its induction suppresses tumor progression and proliferation (10). However, DKK4 is overexpressed in pancreatic cancer, ovarian cancer, gastric cancer, and non-small cell lung cancer. DKK4 activates the MAPK signaling pathway to promote pancreatic cancer progression by regulating VAV3 (11) and promotes ovarian cancer metastasis by activating JNK (7, 12). In CRC, a few studies with limited samples revealed that DKK4 was upregulated in cancer (7, 13), but little is known about the association of DKK4 expression and CRC prognosis, and the molecular mechanism remains unclear.

In the present study, we found that DKK4 is highly expressed among 23 colorectal cancer cell lines and 229 CRC tissues, and high DKK4 expression correlates with a longer survival time of CRC patients. Downregulation of DKK4 promotes the migration and invasion of CRC cells both *in vitro* and *in vivo*. To elucidate the specific mechanism, we found that *rh*Wnt3a activated DKK4 expression, and overexpression of DKK4 inhibited Frizzled 6 (FZD6) and AKT2/s552-β-catenin, forming a negative feedback loop that restrained overactivation of Wnt/β-catenin signaling. Moreover, the expression and function of DKK4 was directly inhibited by miR-450b-5p and miR-299-3p. Overall, our studies demonstrated that DKK4 inhibits tumor metastasis in CRC and uncovered a novel regulatory mechanism through the Wnt3a/DKK4/AKT/s552 β-catenin negative feedback loop in CRC.

## Results

### DKK4 expression is increased in human CRC cells and tissues

**Figure 1:**
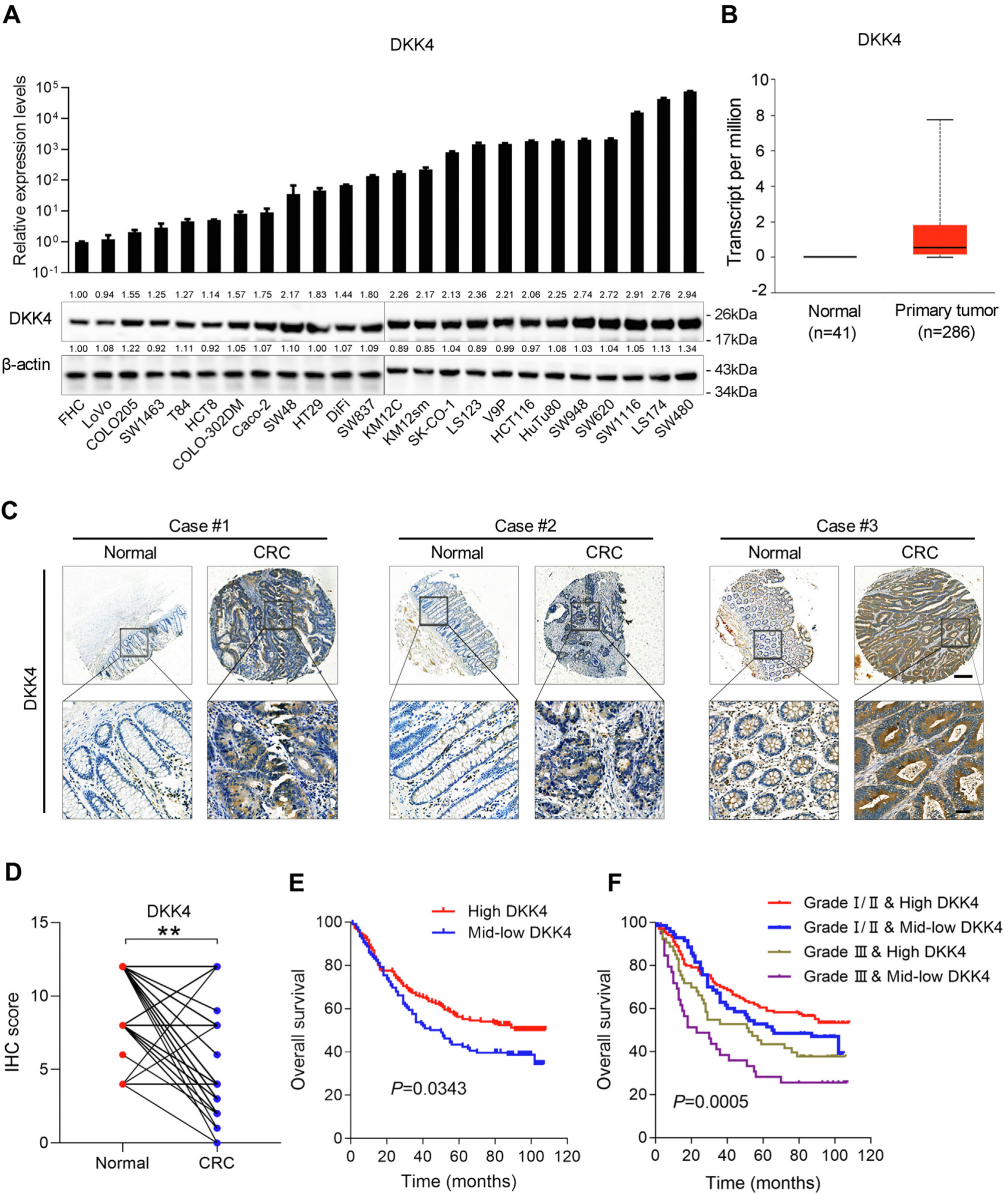
**DKK4 is upregulated in CRC cells and tissues.***A*, the mRNA and protein expression of DKK4 in 23 colorectal cancer cells and FHC measured by qRT‒PCR and Western blot, respectively. *B*, TCGA expression data for DKK4 in normal and primary CRC tissues. *C*, representative images of DKK4 expression in primary CRC tissues and adjacent nontumor tissues detected by immunohistochemistry (IHC) staining. The scale bars represent 500 μm (*upper*) and 50 μm (*lower*). *D*, analysis of the IHC score of DKK4 in primary CRC tissues and adjacent nontumor tissues (n = 229). ∗∗*p* < 0.01. *E*, survival curve of patients in the high DKK4 expression group and medium-low DKK4 expression group in the tissue microarray (n = 293). *F*, survival curve of patients in the high DKK4 expression and medium-low DKK4 expression group between pathological grade III and grade I/II (n = 293). CRC, colorectal cancer; qRT‒PCR, quantitative real-time PCR.

To investigate the DKK4 expression pattern in CRC, we detected DKK4 mRNA levels by quantitative real-time PCR (qRT‒PCR) and protein levels by Western blot in 23 colorectal cancer cell lines and an immortalized human normal intestinal epithelial cell line (FHC). The DKK4 expression level in colorectal cancer cells was significantly higher than that in FHC cells (Fig. 1*A*). Then, we investigated the expression of DKK4 in the TCGA dataset and found that the expression of DKK4 was significantly increased in CRC tissues compared with normal tissues (Fig. 1*B*). Immunohistochemistry (IHC) was conducted in three independent CRC tissue microarrays containing 293 cancer lesions. We found that the DKK4 staining intensity was significantly increased in 229 paired primary CRC tissues compared with adjacent normal tissues (Fig. 1, *C* and *D*). There were no significant differences in sex, age, tumor location, or clinical stage between the high and medium-low DKK4 expression groups (Table S2). Kaplan‒Meier analysis indicated that the survival of CRC patients in the high DKK4 group was longer than that of patients in the medium-low DKK4 expression group (Fig. 1*E*). Further analysis showed that the DKK4 high expression group had a longer survival time than the medium-low expression group, especially in pathological grade I/II group compared to that of grade III (Fig. 1*F*).

### DKK4 inhibits CRC cell migration and invasion *in vitro*

**Figure 2:**
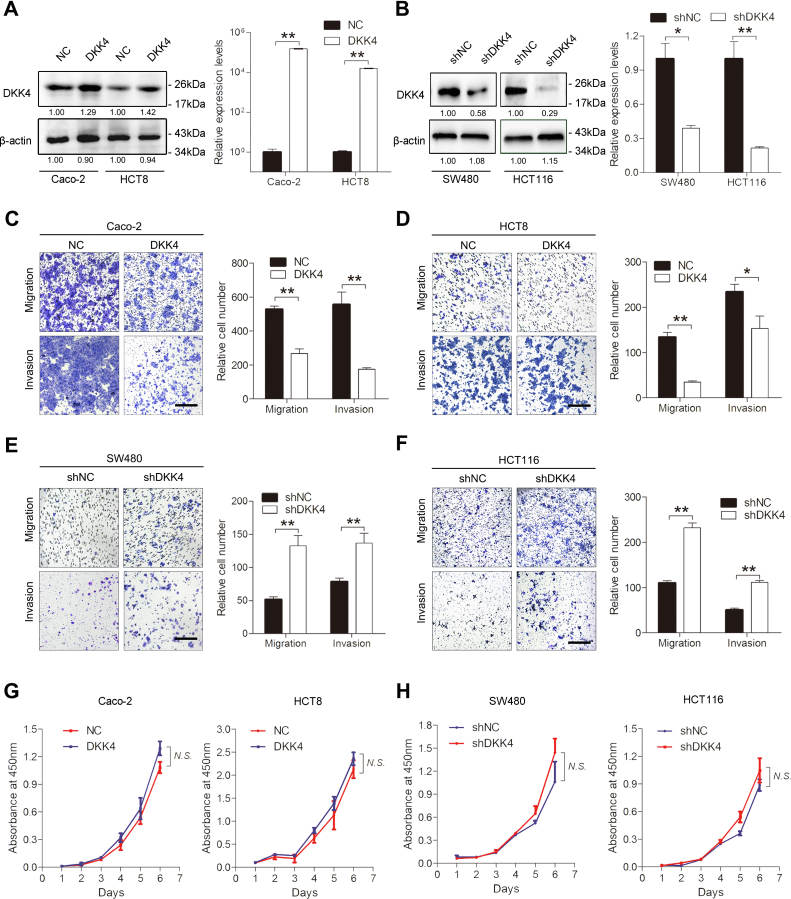
**DKK4 inhibits CRC cell migration and invasion.***A*, DKK4 overexpression models were successfully constructed, as detected by Western blot and qRT‒PCR. ∗∗*p* < 0.01. *B*, DKK4 knockdown models were successfully constructed, as detected by Western blot and qRT‒PCR. ∗*p* < 0.05, ∗∗*p* < 0.01. *C* and *D*, transwell assays showing the migration and invasion abilities of Caco-2 (*C*) and HCT8 (*D*) cells infected with LV-DKK4 and negative controls. The scale bars represent 200 μm. ∗*p* < 0.05, ∗∗*p* < 0.01. *E* and *F*, transwell assays showing the migration and invasion abilities of SW480 (*E*) and HCT116 (*F*) cells infected with LV-shDKK4 and negative controls. The scale bars represent 200 μm. ∗∗*p* < 0.01. *G*, the cell proliferation capacity of DKK4-overexpressing Caco-2 and HCT8 cells detected by CCK-8 assay. N.S., not significant. *H*, the cell proliferation capacity of DKK4-knockdown SW480 and HCT116 cells detected by CCK-8 assay. N.S., not significant. CCK-8, cell counting kit-8; CRC, colorectal cancer; LV-DKK4, Lentivirus carrying the human DKK4 gene; qRT‒PCR, quantitative real-time PCR.

To further explore the roles of DKK4 in CRC, we established gain-of-function models of DKK4 in Caco-2 and HCT8 cells through lentivirus infection and established loss-of-function models of DKK4 in SW480 and HCT116 cells using a lentiviral vector carrying shRNA (Fig. 2, *A* and *B*). Transwell assays showed that DKK4 overexpression decreased the migration and invasion of Caco-2 and HCT8 cells (Fig. 2, *C* and *D*). The migration and invasion ability of Caco-2 in DKK4 overexpression group decreased to 51.9% and 31.7% respectively, compared to NC group. And the migration and invasion ability of HCT8 in DKK4 overexpression group decreased to 26.1% and 65.6%, respectively. Conversely, downregulation of DKK4 expression significantly enhanced the migration and invasion of SW480 and HCT116 cells (Fig. 2, *E* and *F*). The migration and invasion ability of SW480 in DKK4-downregulated group increased to 2.57 and 1.84 fold respectively, compared to NC group. And the migration and invasion ability of HCT116 in DKK4-downregulated group increased to 2.11 and 2.21 fold, respectively. However, DKK4 did not affect cell proliferation (Fig. 2, *G* and *H*). These results indicate that DKK4 inhibits CRC cell migration and invasion but does not influence cell proliferation *in vitro*.

### DKK4 inhibits CRC metastasis *in vivo*

**Figure 3:**
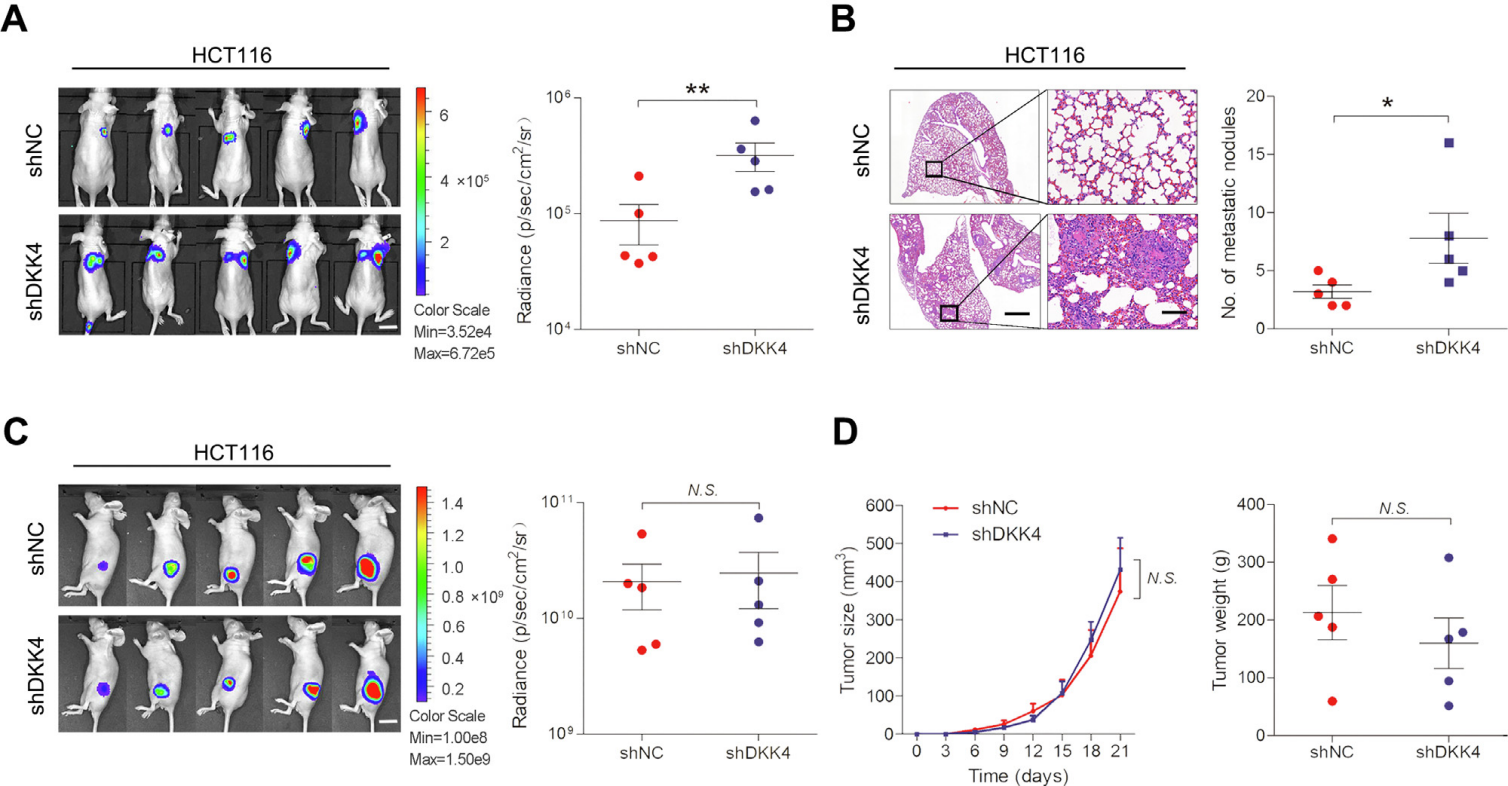
**DKK4 inhibits CRC cell metastasis but not proliferation *in vivo*.***A*, representative bioluminescence images of tumors in nude mice are shown after tail vein injection of DKK4-knockdown HCT116 cells and control cells (n = 5). The scale bars represent 20 mm. ∗∗*p* < 0.01. *B*, H&E staining of lung tissues and the number of metastases from the different groups are shown. The scale bars represent *left*, 1000 μm; *right*, 100 μm. ∗*p* < 0.05. *C*, representative bioluminescence images of the tumors are shown after the subcutaneous injection of DKK4-knockdown HCT116 cells or control cells in nude mice (n = 5). The scale bars represent 20 mm. N.S., not significant. *D*, the tumor volumes were measured every 3 days. The tumor weights were collected at 21 days and compared in nude mice subcutaneously injected with DKK4-knockdown HCT116 cells and control cells (n= 5). N.S., not significant. CRC, colorectal cancer.

To further validate the effect of DKK4 on metastasis *in vivo*, HCT116-LV-shDKK4 and control cells were injected into nude mice via the tail vein. After 5 weeks, *in vivo* bioluminescence imaging showed that the luminescence intensities in the lungs were substantially higher in mice injected with HCT116-LV-shDKK4 cells than in mice in the control group (Fig. 3*A*). The luminescence intensities in the lungs were 3.7-fold in mice injected with HCT116-LV-shDKK4 cells than control group. The number of metastatic nodules in the lungs was also increased in the HCT116-LV-shDKK4 cell group (Fig. 3*B*). HCT116-LV-shDKK4 and the control cells were subcutaneously injected into nude mice to observe tumor growth. Downregulation of DKK4 expression showed no impact on the luminescence intensity, growth rate, or tumor weight between the two groups (Fig. 3, *C* and *D*). These results demonstrate that DKK4 inhibited CRC metastasis *in vivo* but exhibited no effect on tumorigenesis.

### DKK4 regulates Wnt/β-catenin and AKT signaling in CRC cells

**Figure 4:**
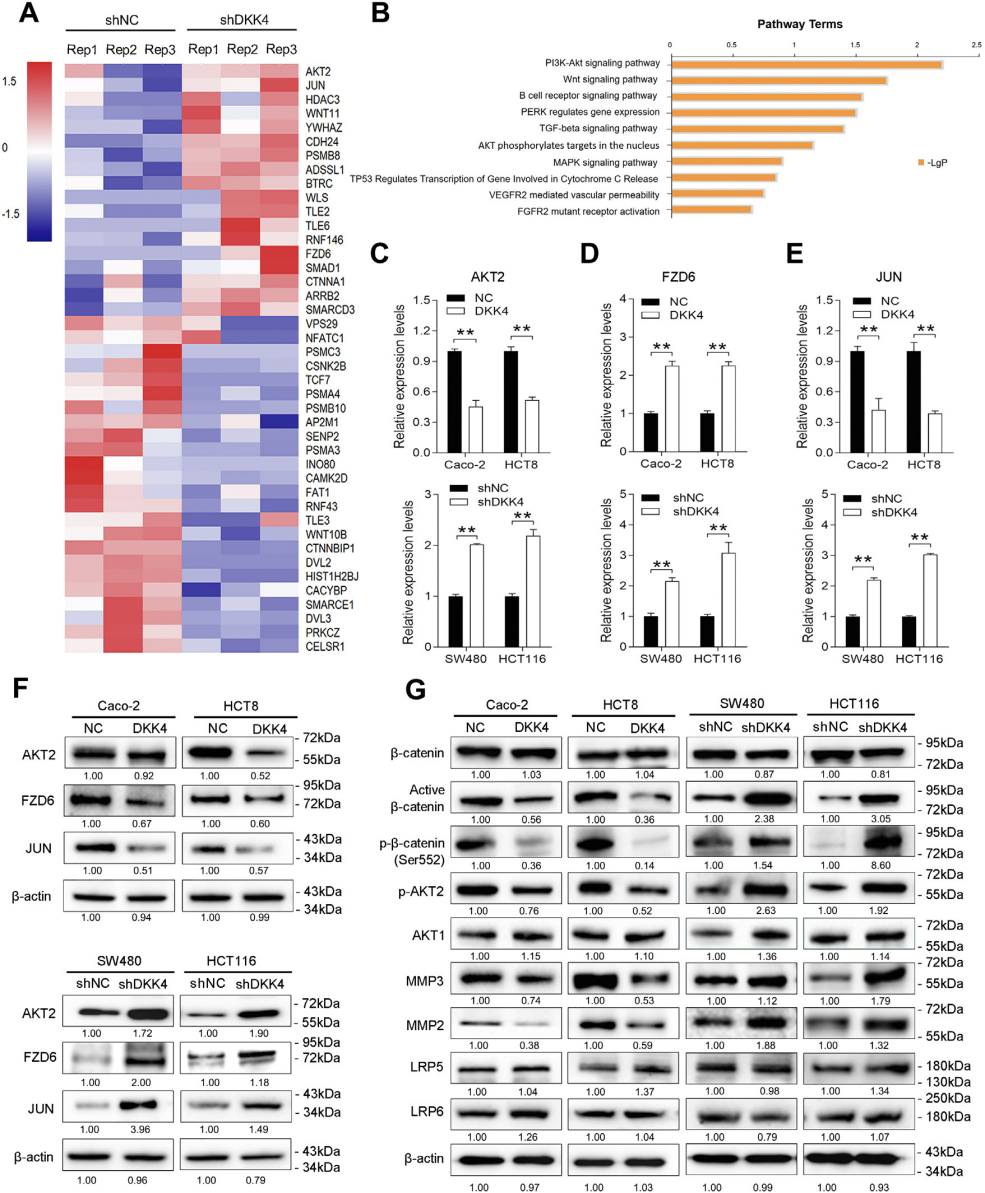
**DKK4 regulates Wnt/β-catenin and AKT signaling in CRC cells.***A*, heatmap showing that 18 genes were upregulated and 24 genes were downregulated at the mRNA level after DKK4 knockdown in HCT116 cells. The cut-off value was a twofold expression change, and the scale bar shows color-coded differences in expression from the mean. *B*, the enriched pathways analyzed by KEGG database. *C*, AKT2 expression was measured by qRT‒PCR in Caco-2 and HCT8 cells infected with LV-DKK4 or negative controls (above) and in SW480 and HCT116 cells infected with LV-shDKK4 or negative controls (below). ∗∗*p* < 0.01. *D*, FZD6 expression was measured by qRT‒PCR in Caco-2 and HCT8 cells infected with LV-DKK4 or negative controls (above) and in SW480 and HCT116 cells infected with LV-shDKK4 or negative controls (below). ∗∗*p* < 0.01. *E*, JUN expression was measured by qRT‒PCR in Caco-2 and HCT8 cells infected with LV-DKK4 or negative controls (above) and in SW480 and HCT116 cells infected with LV-shDKK4 or negative controls (below). ∗∗*p* < 0.05. *F*, Western blot analysis of the expression of AKT2, FZD6, and JUN in Caco-2 and HCT8 cells infected with LV-DKK4 or negative controls (above) and in SW480 and HCT116 cells infected with LV-shDKK4 or negative controls (below). *G*, Western blot analysis of the expression of Wnt and AKT signaling- and metastasis-related proteins in Caco-2 and HCT8 cells infected with LV-DKK4 or negative controls and in SW480 and HCT116 cells infected with LV-shDKK4 or negative controls. CRC, colorectal cancer; FZD6, Frizzled 6; LV-DKK4, Lentivirus carrying the human DKK4 gene; qRT‒PCR, quantitative real-time PCR.

To investigate the underlying mechanism by which DKK4 inhibits tumor metastasis in CRC, we performed RNA-seq to identify potential DKK4-regulated molecules. Using a twofold expression change as a cut-off value for differentially expressed genes, we found that 18 genes were significantly upregulated and 24 genes were significantly downregulated at the mRNA level after DKK4 knockdown in HCT116 cells (Fig. 4*A*). The enrichment analysis of signal pathways indicated that the main enrichment were in PI3K/AKT and Wnt signaling pathways (Fig. 4*B*). Among them, AKT2, FZD6, and JUN, which play important roles in AKT and Wnt signaling (14, 15, 16), were significantly increased. qRT‒PCR and Western blot confirmed that AKT2, FZD6, and JUN expression levels were decreased after DKK4 overexpression in Caco-2 and HCT8 cells and increased after DKK4 knockdown in SW480 and HCT116 cells (Fig. 4, *C*–*F*). Western blot analysis indicated that overexpression of DKK4 in Caco-2 and HCT8 cells led to a great decrease in the total expression and phosphorylation of AKT2. Both active AKT1 and AKT2 were able to phosphorylate β-catenin at Ser552 (17). As expected, active β-catenin, phospho-β-catenin Ser552, MMP2, and MMP3 were also decreased in DKK4-overexpressing Caco-2 and HCT8 cells (Fig. 4*G*). In contrast, the expression levels of total and phosphorylated AKT2, active β-catenin, and s552-β-catenin as well as MMP2 and MMP3, the downstream target genes of AKT and Wnt signaling, were significantly increased when DKK4 was knocked down in HCT116 and SW480 cells (Fig. 4*G*). However, the expression of total β-catenin, AKT1, LRP5, and LRP6 showed no significant difference between DKK4 upregulated or downregulated cells and control cells (Fig. 4*G*).

**Figure 5:**
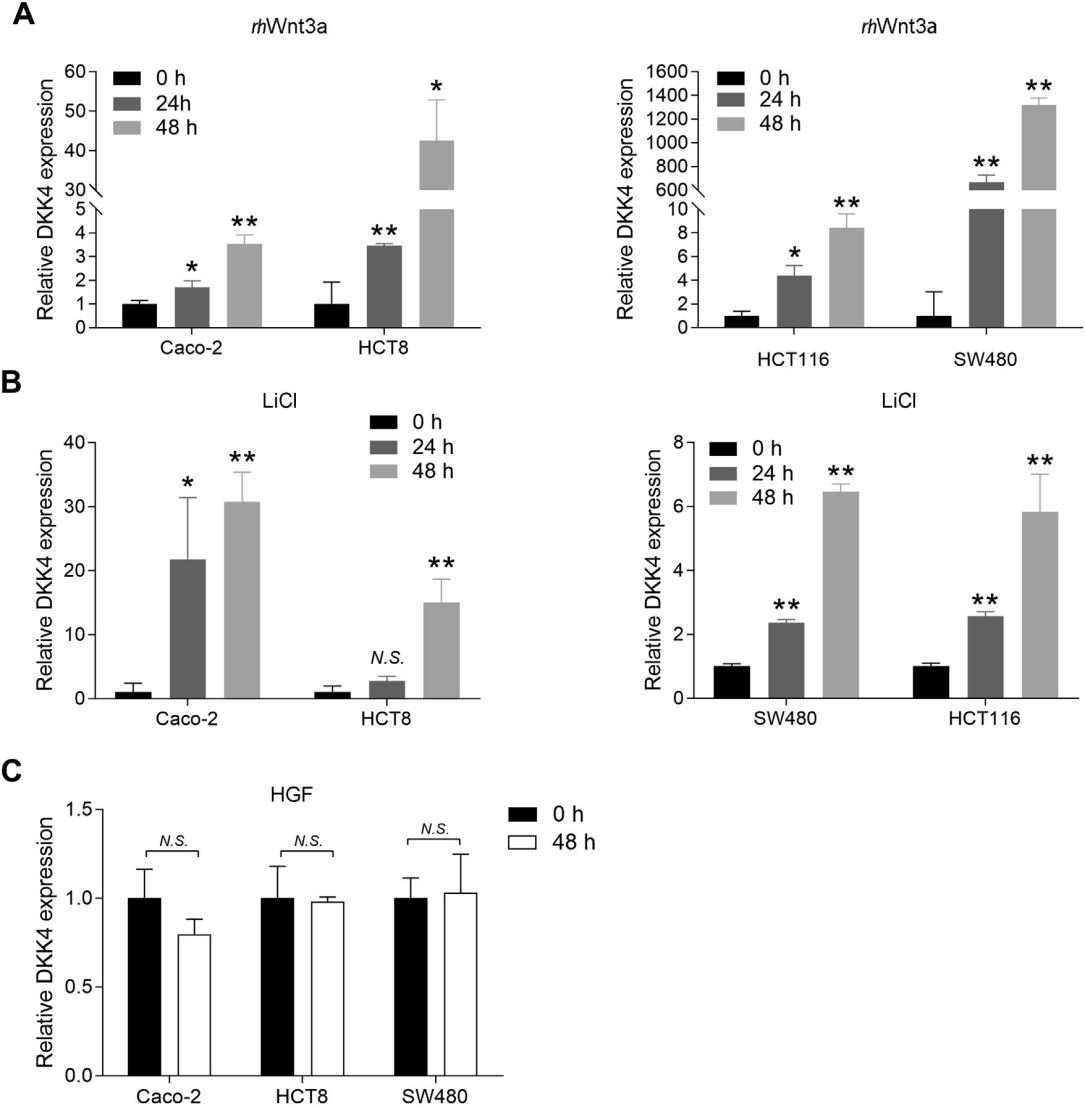
**Wnt signaling activation induced DKK4 expression.***A*, expression of DKK4 in Caco-2, HCT8, SW480, and HCT116 cells stimulated with *rh*Wnt3a (50 ng/ml). ∗*p* < 0.05, ∗∗*p* < 0.01. *B*, expression of DKK4 in Caco-2, HCT8, SW480, and HCT116 cells stimulated with LiCl (20 mmol/l). ∗*p* < 0.05, ∗∗*p* < 0.01. *C*, expression of DKK4 in Caco-2, HCT8, and SW480 cells stimulated with HGF (50 ng/ml). N.S., not significant; HGF, hepatocyte growth factor.

The antimetastatic effect of DKK4 seems paradoxical to its upregulation in CRC. We thus examined the cause of DKK4 upregulation in CRC cells. Since DKK4 inhibits Wnt signaling activity and DKK4 has been reported to be a transcriptional target of Wnt signaling (10, 18), we hypothesized that DKK4 represents a feedback mechanism of aberrant activation of Wnt signaling in the development of CRC. To test this hypothesis, we stimulated CRC cell lines with recombinant human Wnt3a (*rh*Wnt3a), which is an important ligand of Wnt signaling. We found that upon stimulation with *rh*Wnt3a, DKK4 mRNA expression was significantly increased at 24 and 48 h (Fig. 5*A*). As LiCl is the Wnt agonist, we stimulated cell lines with LiCl and found that the expression of DKK4 was significantly increased at 24 and 48 h (Fig. 5*B*). However, no change was observed when cells were stimulated with hepatocyte growth factor (HGF) (Fig. 5*C*). These data suggested that DKK4 overexpression in CRC might be a feedback loop of Wnt signaling triggered by growth factors in the microenvironment, such as Wnt3a. These results suggest that Wnt3a induced DKK4 expression and that DKK4 inhibited CRC progression by decreasing the activities of AKT and Wnt signaling.

### miR-450b-5p and miR-299-3p promote CRC cell migration and invasion by directly suppressing DKK4

**Figure 6:**
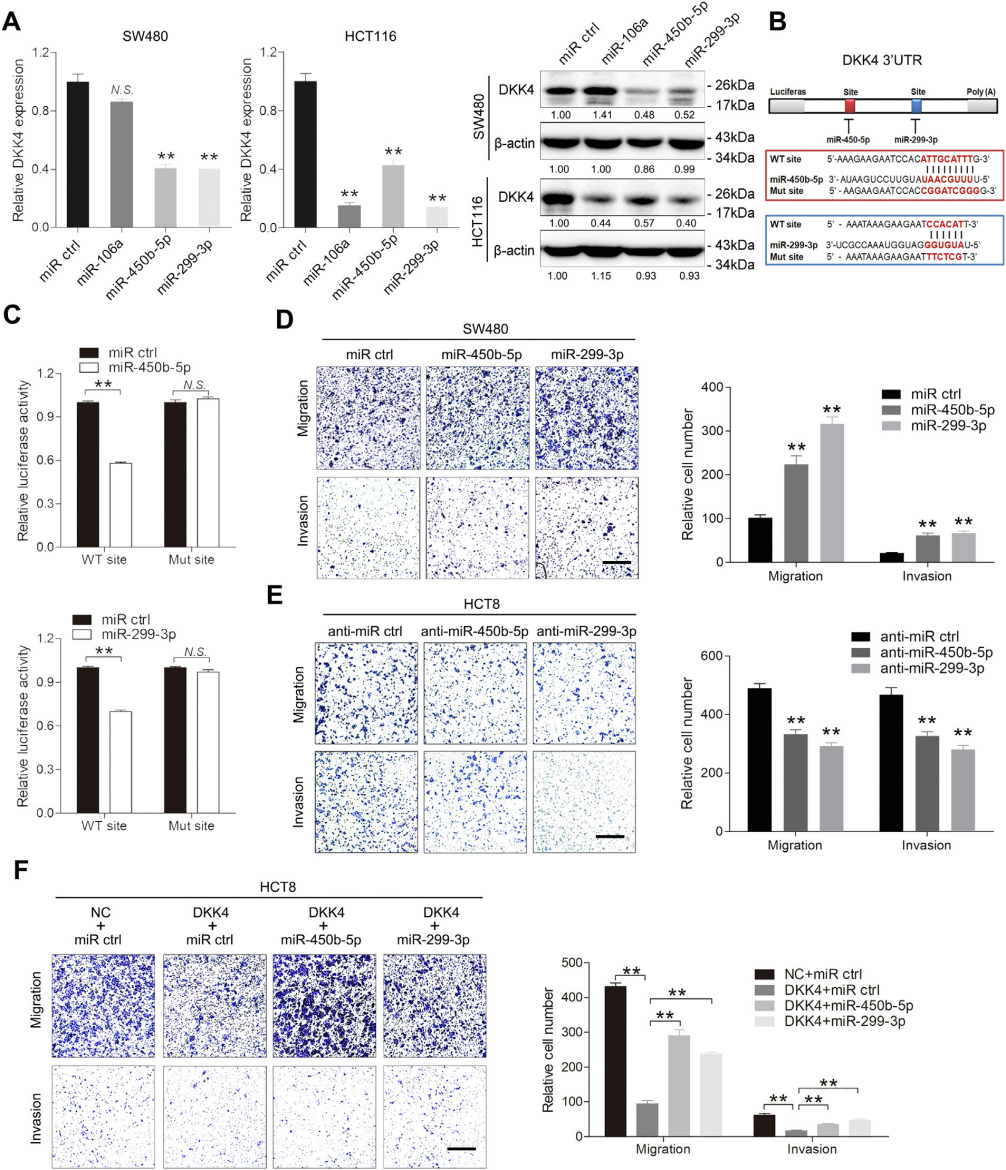
**miR-450b-5p and miR-299-3p directly target DKK4 in CRC cells.***A*, qRT‒PCR and Western blot of DKK4 expression in SW480 and HCT116 cells transfected with miR-106a, miR-450b-5p, or miR-299-3p mimics and their controls. N.S., not significant. ∗∗*p* < 0.01. *B*, a schematic representation of the predicted duplex sequences between the DKK4 3′-UTR and miR-450b-5p and miR-299-3p. Mutations were designed at the predicted miR-450b-5p- and miR-299-3p-binding sites. *C*, the DKK4 WT and mutant reporter plasmids were cotransfected with miR-450b-5p (above), miR-299-3p (below) mimic, and the controls. Luciferase activity values were measured and analyzed. N.S., not significant. ∗∗*p* < 0.01. *D*, transwell assay showing the migration and invasion abilities of SW480 cells transfected with miR-450b-5p and miR-299-3p mimics or the corresponding negative control. The scale bars represent 200 μm. ∗∗*p* < 0.01. *E*, transwell assay showing the migration and invasion abilities of HCT8 cells transfected with miR-450b-5p and miR-299-3p inhibitors or the corresponding negative control. The scale bars represent 200 μm. ∗∗*p* < 0.01. *F*, transwell assay showing the migration and invasion abilities of HCT8 cells transfected with LV-DKK4 in combination with or without miR-450b-5p and miR-299-3p mimics. The scale bars represent 200 μm. ∗∗*p* < 0.01. CRC, colorectal cancer; LV-DKK4, Lentivirus carrying the human DKK4 gene; qRT‒PCR, quantitative real-time PCR.

**Supplementary Figure 1:**
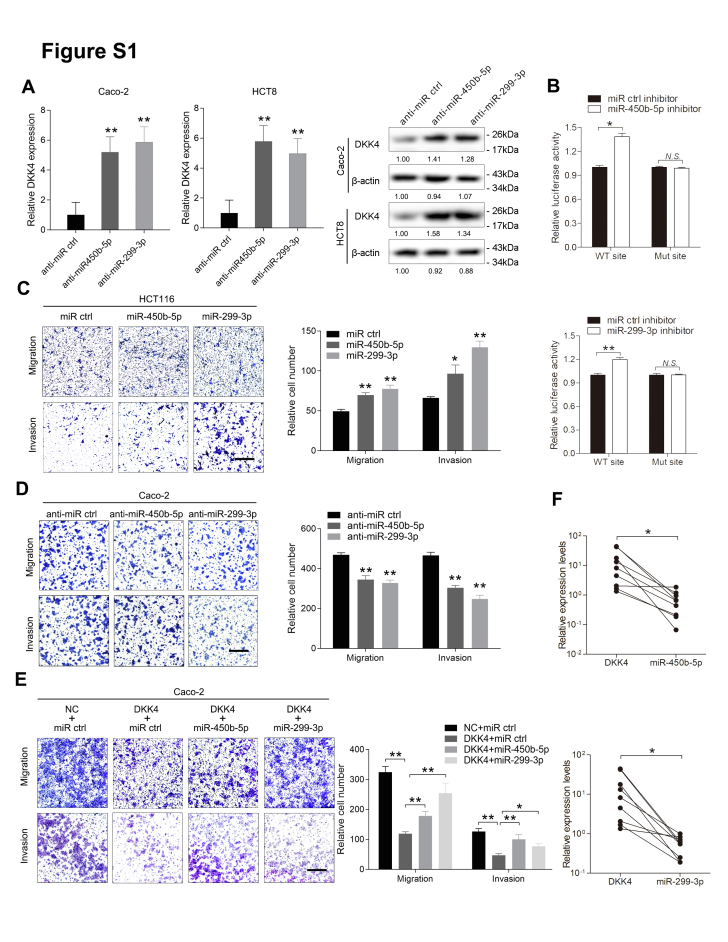
**miR-450b-5p and miR-299-3p promote CRC cell metastasis and invasion by directly suppressing DKK4 expression.***A*, qRT‒PCR and Western blot of DKK4 expression in Caco-2 and HCT8 cells transfected with miR-450b-5p or miR-299-3p inhibitors and their controls. ∗∗*p* < 0.01. *B*, the DKK4 wild-type and mutant reporter plasmids were cotransfected with miR-450b-5p (above), miR-299-3p (below) inhibitor or the control. Luciferase activity values were measured. N.S., not significant. ∗*p* < 0.05, ∗∗*p* < 0.01. *C*, transwell assay and corresponding data analyses showing the migration and invasion abilities of HCT116 cells transfected with miR-450b-5p or miR-299-3p mimics or the corresponding negative control. Scale bar: 200 μm. ∗*p* < 0.05, ∗∗*p* < 0.01. *D*, transwell assay and corresponding data analyses showing the migration and invasion abilities of Caco-2 cells transfected with miR-450b-5p or miR-299-3p inhibitors or the corresponding negative control. Scale bar: 200 μm. ∗∗*p* < 0.01. *E*, transwell assay showing the migration and invasion abilities of Caco-2 cells transfected with LV-DKK4 in combination with or without miR-450b-5p or miR-299-3p mimics. Scale bars: 200 μm. ∗*p* < 0.05, ∗∗*p* < 0.01. *F*, the associations between DKK4 levels and miR-450b-5p (above) or miR-299-3p (below) expression in 9 paired CRC tissues measured by qRT‒PCR. ∗*p* < 0.05.

Low DKK4 expression correlated with poor survival in CRC patients (Fig. 1*E*). miRNAs are important regulators that repress gene expression at the posttranscriptional level. We next investigated whether DKK4 was regulated by miRNAs. To this end, bioinformatics prediction was used to identify potential miRNAs that target DKK4. The miRWalk database identified that miR-299-3p, miR-450b-5p, miR-106a, miR-203, miR-129-5p, and miR-507 may bind to the DKK4 3′-UTR sequence. Among these miRNAs, miR-299-3p, miR-450b-5p, and miR-106a, which were reported to be involved in tumorigenesis (19, 20, 21), ranked in the top three calculated by both the total context ++ score and aggregated preferentially conserved targeting algorithms. Mimics of the three miRNAs were transfected into SW480 and HCT116 cells. qRT‒PCR and Western blot confirmed that miR-450b-5p and miR-299-3p inhibited DKK4 expression (Fig. 6*A*). Inhibitors of miR-450b-5p and miR-299-3p were transfected into Caco-2 and HCT8 cells. qRT‒PCR and Western blot confirmed that lower miR-450b-5p and miR-299-3p levels increased DKK4 expression (Fig. S1*A*). To assess whether miR-450b-5p and miR-299-3p directly bind to the 3′-UTR of DKK4, we performed dual-luciferase reporter assays. Both miR-450b-5p and miR-299-3p affected the luciferase activity of the DKK4 WT 3′-UTR reporter construct, whereas this effect was abolished by the introduction of a mutation into the miR-299-3p- or miR-450b-5p-binding sequence, respectively (Figs. 6, *B* and *C* and S1*B*). These results indicate that miR-450b-5p and miR-299-3p downregulate DKK4 expression by directly targeting its 3′-UTR.

To verify whether DKK4 is the functional target of miR-450b-5p and miR-299-3p, we overexpressed miR-450b-5p and miR-299-3p in SW480 and HCT116 cells, inhibited their levels in Caco-2 and HCT8 cells, and examined their effects on migration and invasion. Transwell assays revealed that miR-450b-5p or miR-299-3p overexpression promoted the migration and invasion of SW480 and HCT116 cells (Figs. 6*D* and S1*C*). Conversely, their downregulation decreased the migration and invasion of Caco-2 and HCT8 cells (Figs. S1*D* and 6*E*). Furthermore, we cotransfected the DKK4-3′-UTR-overexpressing plasmid and miR-450b-5p or miR-299-3p mimic in HCT8 and Caco-2 cells. We found that transfection of miR-450b-5p or miR-299-3p into DKK4-overexpressing cells abrogated the inhibitory effect of DKK4 on migration and invasion (Figs. 6*F* and S1*E*). We also found that miR-450b-5p and miR-299-3p expression was inversely correlated with DKK4 levels in nine paired primary CRC tissues and their adjacent normal tissues (Fig. S1*F*). These findings suggest that DKK4 in CRC is regulated by miR-450b-5p and miR-299-3p.

## Discussion

In the present study, we found that DKK4 expression was significantly upregulated in primary and metastatic CRC tissues compared with adjacent tissues. Several studies reported the upregulation of DKK4 in CRC cells and tissues (7), but they included limited cell lines and tissue samples. Our studies confirmed the upregulation of DKK4 in CRC by enrolling 23 CRC cell lines and tissue microarrays containing 229 CRC cases in total. DKK4 expression was closely correlated with poor prognosis of gastrointestinal stromal tumors (22, 23). However, there have been no studies on DKK4 expression in correlation with the prognosis of CRC patients. In our study, we found that high DKK4 expression is correlated with prolonged survival in colorectal cancer patients. The DKK4 high expression group had a longer survival time than the medium-low expression group, especially in pathological grade I/II group compared to that of grade III.

The dysregulation of DKK4 plays a significant role in the process of initiation and metastasis in various cancer types. Studies have demonstrated the tumor-promoting role of DKK4 in renal cell carcinoma, non-small cell lung cancer, and ovarian cancer. In contrast, DKK4 is commonly considered a tumor suppressor in gastrointestinal cancers such as liver cancer and gastric cancer. These data indicate a cell- or tissue-context function of DKK4 in malignant cancer progression (7). In CRC, previous studies revealed that DKK4 increased angiogenic potential but showed a controversial role in cell migration (24). Our work indicated the tumor suppressive role of DKK4 in CRC metastasis and showed that DKK4 had no effect on cell proliferation. The Wnt/β-catenin signaling pathway is a highly conserved pathway in embryonic development, and its aberrant activation is tightly linked with the malignant progression of many human cancers (25). Previous study indicated that cells with mutations in APC or β-catenin still depend on Wnt ligands and their secretion for a sufficient level of β-catenin signaling (26). Our findings are consistent with this report and we provide evidence that DKK4 functions in multiple CRC cell lines with different genetic background.

DKK family members such as DKK1 and DKK2 have been shown to bind LRP5/6 with high affinities and inhibit their expression (7), but we did not find any change of LRP5/6 expression after DKK4 inhibition, indicating DKK4 does not function through inhibiting LRP5/6. To explore the further mechanism, the enrichment analysis of signal pathways from RNA-seq data indicated that the main enrichment were in PI3K/AKT and Wnt signaling pathways. Among them, qRT‒PCR and Western blot analysis confirmed that FZD6, AKT2, and JUN showed significant changes after DKK4 knockdown in SW480 and HCT116 cell lines, whereas other molecules such as CTNNA1 exhibited no obvious change. FZD6 is known as an upstream receptor for Wnt protein in β-catenin signaling activation， which can promote CRC progression through the activation of the Wnt/β-catenin pathway (27). AKT phosphorylates β-catenin at Ser552 to induce the accumulation of β-catenin in the nucleus and enhance its transcriptional activity (14, 28). c-Jun is a nuclear protein, which might be involved in the canonical Wnt signaling pathway and act downstream of β-catenin stabilization (29). We found that DKK4 suppress the Wnt/β-catenin pathway not only by directly inhibiting FZD6 expression but also by inhibiting the expression of AKT2 and phospho-AKT2, leading to the suppression of ser552 β-catenin. Metalloproteinases are target genes of the Wnt/β-catenin and AKT pathways. We further confirmed that DKK4 could inhibit the expression of MMP2 and MMP3.

Paradoxically, why is DKK4 increased in the CRC metastasis setting, while it plays an inhibitory role on the Wnt/β-catenin pathway? We found that *rh*Wnt3a and LiCl could increase DKK4 expression. Since Wnt3a is an abundant growth factor in the CRC tumor microenvironment that is conducive to metastasis, this finding provides a rationale for why DKK4 is upregulated in tumors but plays a suppressive role. Altogether, our study indicated that *rh*Wnt3a or LiCl increases DKK4 expression, while overexpressed DKK4 in turn suppresses Wnt signaling, forming a negative feedback loop to limit overactivation of the Wnt/β-catenin pathway in CRC.

miRNAs are important epigenetic regulators that modulate the expression of critical cancer-related genes and thereby function as oncogenes or tumor suppressor genes (30). To date, no studies have reported specific miRNAs that regulate the expression of DKK4. In this study, we employed bioinformatics analyses and subsequent experiments to identify the regulatory miRNAs of DKK4 in CRC cells. MiR-450b-5p and miR-299-3p were predicted and demonstrated to target DKK4, and an inverse correlation was observed between miR-450-5p or miR-299-3p and DKK4 in CRC tissues. The decreased metastatic ability caused by overexpression of DKK4 was partially eliminated by the ectopic expression of miR-450b-5p and miR-299-3p. Overall, our results showed that miR-450b-5p and miR-299-3p directly targeted DKK4 in CRC.

In summary, we found for the first time that DKK4 is upregulated in CRC but plays a suppressive role in tumor metastasis. Regarding the mechanism, we identified a novel Wnt3a/DKK4/AKT/s552 β-catenin negative feedback regulatory axis that may limit Wnt signaling from overactivation in CRC. Additionally, we found that the expression of DKK4 was regulated by miR-450b-5p and miR-299-3p. These results suggest that DKK4 serves as a promising prognostic marker and that the restoration of DKK4 expression may be an effective strategy for the treatment of CRC.

## Experimental procedures

### Cell culture

The human CRC cell lines LoVo, COLO205, SW1463, T84, HCT8, COLO-320DM, Caco-2, SW48, HT29, DiFi, SW837, KM12C, KM12sm, V9P, SK-CO-1, LS123, HCT116, HuTu80, SW948, SW630, SW1116, LS174, and SW480 and the human normal colonic epithelial cell lines FHC and 293T were obtained from the American Type Culture Collection. The origin and mutation status of the Caco-2, HCT8, SW480, and HCT116 cell lines are shown in Table S1. All cells were cultured in Dulbecco’s modified Eagle’s medium (Gibco) with 10% fetal calf serum (Gibco), 1% antibiotic-antimycotic (Gibco), 1% L-glutamine, and 1% MEM nonessential amino acids (Gibco) at 37 °C in 5% CO_2_. The complete medium used for culturing CC-CR cells contained 3 μg/ml cetuximab (Merck).

### Tissue specimens

Three colorectal cancer tissue microarrays (HCoLA180Su10, HCoLA180Su15, and HCoLA180Su17) were purchased from the Outdo Biotech Company, which includes 229 paired human CRC tissues and the corresponding adjacent nontumorous colorectal tissues and in addition, 64 cases only with primary cancer lesions. Nine primary CRC and matched adjacent normal tissue samples were collected from patients who underwent CRC surgery at Xijing Hospital of Digestive Diseases. The samples were frozen in liquid nitrogen. All samples were clinically and pathologically confirmed to be correctly labeled. All patients provided informed consent, and this study was approved by Xijing Hospital’s Protection of Human Subjects Committee.

### Immunohistochemistry

IHC staining for DKK4 was performed on tissue microarrays. The tissue microarrays were first baked on a 65 °C toaster for 2.5 h, deparaffinized, subjected to antigen retrieval with sodium citrate buffer at a pH of 6.0 and endogenous peroxidase inactivation, and then incubated overnight at 4 °C with a rabbit anti-human DKK4 primary antibody (Abcam). The slides were then incubated with secondary antibodies and visualized using 3,3-diaminobenzidine tetrahydrochloride plus. The IHC scoring criteria were as follows: “positive staining rate” score: ≤1% (0), 2 to 25% (1), 26 to 50% (2), 51 to 75% (3), and ≥75% (4); “staining intensity” score: negative (0), weak (1), moderate (2), and strong (3); and histological score = intensity score × percentage score. An overall score of 0 to 12 was graded as negative (−, score: 0), weak (+, score: 1–4), moderate (++, score: 5–8), or strong (+++, score: 9–12).

### Quantitative real-time PCR

Total RNA was extracted from cultured cells and surgically resected fresh-frozen CRC tissues with QIAzol Reagent and purified using a miRNAeasy Kit (Qiagen) according to the manufacturer’s instructions. For miRNA detection, total RNA was used for complementary DNA synthesis with a TaqMan miRNA Reverse Transcription Kit (Applied Biosystems). For mRNA detection, total RNA was reverse transcribed with a QuantiTect Reverse Transcription Kit (Qiagen), and the double-stranded cDNA was amplified by real-time PCR by SYBR Premix Ex Taq (Takara). Real-time PCR was performed in triplicate using SYBR Premix Ex TaqTM II (Takara) with the CFX96 QPCR Detection System (Bio-Rad). The primers specific to mature miR-450b-5p and miR-299-3p were purchased from RiboBio. The primers for the other genes of interest were synthesized by Takara. U6 and GAPDH were used as internal controls for miRNA and DKK4, respectively. The 2^−ΔΔCT^ method was used to determine the fold changes in the mRNA levels between each sample and the reference sample. The PCR primers were as follows: DKK4-forward 5′-AAGCCGTTCTGTGCTACATG-3′, DKK4-reverse 5′-CTTTTGGGTTGGTTTTCCTG-3′ and GAPDH-forward 5′-GCACCGTCAAGGCTGAGAAC-3′, GAPDH-reverse 5′-TGGTGAAGACGCCAGTGG A-3′.

### Western blot

Cultured CRC cells were lysed in RIPA Lysis Buffer (Beyotime) supplemented with a protease inhibitor and phosphatase inhibitor (Roche). Protein lysates were separated by SDS‒PAGE and transferred onto nitrocellulose membranes (Millipore). The membranes were sequentially blocked with 10% skim milk in TBST (150 mM NaCl, 120 mM Tris–HCl, pH of 7.4, and 0.05% Tween 20), incubated with primary antibodies, and incubated with secondary HRP-conjugated antibodies (Cell Signaling Technology). The antibodies used were as follows: rabbit anti-human DKK4 (Abcam), mouse anti-human β-actin (Sigma‒Aldrich), rabbit anti-human β-catenin, rabbit anti-human nonphospho (active)-β-catenin (Ser33/37/Thr41), rabbit anti-human phospho-β-catenin (Ser552), rabbit anti-human AKT1, AKT2, phospho-AKT2, FZD6, JUN, LRP5, LRP6, MMP2, and MMP3 (Cell Signaling Technology), and goat anti-rabbit or goat anti-mouse IgG (ZSGB). β-actin was used as an internal control. The immunoreactive protein complexes were detected with enhanced chemiluminescence reagents (ZETA) and scanned by the Molecular Imager ChemiDox XRS+ Imaging System with Image Lab software (Bio-Rad).

### Transient transfection and lentivirus infection

Small interfering RNAs against DKK4 (siDKK4), mimics of miR-450b-5p, miR-299-3p and corresponding control oligonucleotides were designed and synthesized by GenePharma. Inhibitors of miR-450b-5p, miR-299-3p, and corresponding control oligonucleotides were designed and synthesized by RIBOBIO. DKK4 siDKK4#1 5′-UUUCUGGUAUUGCAGUCCGTT-3′, siDKK4#2 5′-UGAUUUCUUAAUACUUGGCTT-3′, siDKK4#3 5′-UUUAUGCCCUCUUCUGGAGTT-3′, and the negative control siRNA 5′-ACGUGACACGUUCGGAGAATT-3′ were transfected into HCT116 and SW480 cells, and HCT116 and SW480 cells were transfected with miR-299-3p and miR-450b-5p mimics and a scrambled negative control miRNA. Transfection of the siRNAs and miRNA mimics was performed using DharmaFECT transfection reagent (Thermo Fisher Scientific) according to the manufacturer’s instructions. Lentivirus carrying the human DKK4 gene (LV-DKK4) was purchased from Genepharma, and short hairpin RNA targeting DKK4 (LV-shDKK4) was purchased from GeneChem. An empty lentiviral vector was used as a negative control. After 48 h of infection, CRC cells were screened with 2.5 μg/ml puromycin (MP Biomedicals) for 2 weeks to obtain cell lines with stable infection.

### Cell proliferation

Treated cells were seeded at a density of 1 × 10^3^ per well in 96-well plates. After incubation for 24, 48, 72, 96, and 120 h, a cell counting kit-8 assay (Dojindo) was utilized to examine cell proliferation according to the manufacturer’s instructions. The absorbance was measured at 450 nm using a microplate reader (Thermo Fisher Scientific).

### *In vitro* migration and invasion assays

The cell migration and invasion capabilities were assessed by transwell assay with 8-μm pore, 24-well transwell plates (Corning). For the migration assays, 10 × 10^5^ Caco-2 LV-DKK4, HCT8 LV-DKK4, HCT116-LV-shDKK4, SW480 LV-shDKK4, or corresponding negative control cells were seeded in the top chamber lined with an uncoated membrane (Millipore). In the invasion experiment, 10 × 10^5^ of the aforementioned cells were plated in the upper chamber coated with Matrigel (BD Biosciences). After incubation at 37 °C with 5% CO_2_ for an appropriate duration, cells that had migrated or invaded through the membrane were stained with 0.1% crystal violet and counted under a microscope (Olympus) to determine their relative numbers.

### *In vivo* tumorigenicity and tail vein metastasis assays

Female nude mice aged 4 to 6 weeks were provided by the Experimental Animal Center of the Fourth Military Medical University and housed in pathogen-free conditions. Animal studies were approved by the Fourth Military Medical University Animal Care Committee. All mice were randomized into groups. Luciferase-labeled HCT116-LV-shDKK4 and negative control cells were used in subcutaneous xenograft and tail vein metastasis assays. For *in vivo* tumorigenicity assays, the abovementioned cells were trypsinized, washed, and resuspended in PBS. A total of 5 × 10^6^ cells in 200 μl of PBS were subcutaneously injected into the right flanks of mice (n = 5). The tumor volumes were measured every 3 days. Mice were sacrificed at 4 weeks, and the subcutaneous tumors were dissected for histological examination.

For *in vivo* metastasis assays, a total of 1 × 10^6^ cells in 200 μl of PBS were injected via the tail vein into nude mice (n = 5). Five weeks after injection, the bioluminescence intensity was evaluated using the IVIS Spectrum *in vivo* imaging system (PerkinElmer). Eight weeks after injection, the mice were euthanized, and the lung metastases were dissected for further histological examination.

### Luciferase reporter assay

The indicated cells cultured in 24-well plates were cotransfected with miR-450b-5p or miR-299-3p mimic or negative control and the indicated WT or mutant psiCHECK2-3′UTR plasmids using Lipofectamine 2000 (Thermo Fisher Scientific). Cells were collected and lysed for luciferase assays 48 h after transfection. A dual-luciferase assay (Promega) was used to quantify the luciferase activity. The firefly luciferase activity was normalized to Renilla luciferase activity and presented as the relative luciferase activity. All assays were performed in triplicate.

### Wnt3a, LiCl, and HGF act on CRC cell lines

These Caco-2, HCT8, SW480, and HCT116 cell lines were cultured serum-free for 24 h. Recombinant human *rh*Wnt3a (50 ng/ml) or LiCl (20 mmol/l) or HGF (50 ng/ml) was added in Caco-2, HCT8, SW480 cell lines, and cells at 0 h, 24 h, and 48 h were removed and rinsed with PBS. Then, QIAzol was added to extract RNA.

### Databases

The Cancer Genome Atlas (TCGA; https://cancergenome.nih.gov) datasets were used to detect the expression of DKK4 mRNA in human cancer specimens and normal tissues.

### Statistical analysis

Data collected from at least three independent experiments were analyzed using SPSS 24.0 software. Normally distributed data and non-normally distributed data are presented as the mean ± SEM, and Student’s unpaired *t* test and the Mann‒Whitney U test were used for comparisons between two groups, respectively. Classification data were analyzed using Fisher’s exact test. Frequencies of categorical variables were compared using the χ^2^ test. Spearman’s rank correlation coefficients were calculated to assess mutual associations among clinical results. *p* < 0.05 was considered representative of a significant difference (∗*p* < 0.05 and ∗∗*p* < 0.01).

## Data availability

The data used and/or analyzed in the present study are available from the corresponding author on reasonable request.

## Supporting information

This article contains supporting information.

## Conflict of interest

The authors declare that they have no conflicts of interest with the contents of this article.
