# Supplementary material for: Wnt signaling modulator DKK4 inhibits colorectal cancer metastasis through an AKT/Wnt/β-catenin negative feedback pathway
Source: J Biol Chem. 2022 Sep 29;298(11):102545. doi: 10.1016/j.jbc.2022.102545 (PMC9640985; doi:10.1016/j.jbc.2022.102545)
Supplement: Supplementary Tables 1 and 2 [file mmc1.docx]

**Supplementary Table 1.**

**Mutation status of CRC cell lines used in this study**

| **Cell line** | **APC** | **CTNNB1** | **KRAS** | **BRAF** | **PI3K** | **PTEN** | **TP53** |
| --- | --- | --- | --- | --- | --- | --- | --- |
| **Caco-2** | MUT | MUT | WT | WT | WT | WT | MUT |
| **HCT8** | MUT | MUT | MUT | WT | MUT | WT | WT |
| **SW480** | MUT | WT | MUT | WT | WT | WT | MUT |
| **HCT116** | WT | MUT | MUT | WT | MUT | WT | WT |

WT, wild type; MUT, mutant.

**Supplementary Table 2.**

**Correlation of DKK4 expression and the clinical characteristics of CRC patients**

| **Group** |  | **DKK4 High** | **DKK4 Mid-Low** | **P value** |
| --- | --- | --- | --- | --- |
|  |  | (n=186) | (n=107) |  |
| **Age** |  | 68.21±10.92 | 67.43±10.73 | 0.557 |
| **Gender** | Male | 96.00 | 62.00 | 0.326 |
|  | Female | 90.00 | 45.00 |  |
| **Tumour size** | <5cm | 108 | 60 | 0.143 |
|  | ≥5cm | 78 | 47 |  |
| **Grade** | 1 | 0 | 1 | 0.166 |
|  | 2 | 133 | 68 |  |
|  | 3 | 53 | 38 |  |
| **Location** | Left | 88 | 47 | 0.143 |
|  | Right | 98 | 60 |  |
| **T-stage** | T1 | 1 | 2 | 0.358 |
|  | T2 | 8 | 5 |  |
|  | T3 | 141 | 73 |  |
|  | T4 | 36 | 27 |  |
| **N-stage** | N0 | 109 | 59 | 0.425 |
|  | N1 | 60 | 32 |  |
|  | N2 | 17 | 16 |  |
| **M-stage** | M0 | 178 | 106 | 0.104 |
|  | M1 | 8 | 1 |  |
| **AJCC stage** | I | 9 | 6 | 0.335 |
|  | II | 99 | 53 |  |
|  | III | 70 | 47 |  |
|  | IV | 8 | 1 |  |
